# Render lighting dataset: A collection of rendered images with varied lighting conditions using blender render engines

**DOI:** 10.1016/j.dib.2024.110331

**Published:** 2024-03-16

**Authors:** Khandoker Ashik Uz Zaman, Ashraful Islam, Md Abu Sayed

**Affiliations:** a Department of Computer Science and Engineering, Independent University Bangladesh, Dhaka 1229, Bangladesh; b Center for Computational & Data Sciences, Independent University Bangladesh, Dhaka 1229, Bangladesh

**Keywords:** 3D rendering lighting, Blender, 3D render engines, 3D lighting conditions, 3D materials, Computer graphics, Machine Learning, Image dataset

## Abstract

The quality of datasets is crucial in computer graphics and machine learning research and development. This paper presents the Render Lighting Dataset, featuring 63,648 rendered images of Blender's primitive shapes with various lighting conditions and engines. The images were created using Blender 4.0′s Cycles and Eevee Render Engines, with careful attention to detail in texture mapping and UV unwrapping. The dataset covers six different lighting conditions, including Area Light, Spotlight, Point Light, Tri-Light, HDRI (Sunlight), and HDRI (Overcast), each adjusted using Blender's different options in the Color Management panel. With thirteen unique materials, ranging from Coastal Sand to Glossy Plastic, the dataset provides visual diversity for researchers to explore material properties under different lighting conditions using different render engines. This dataset serves as a valuable resource for researchers looking to enhance 3D rendering engines. Its diverse set of rendered images under varied lighting conditions and material properties allows researchers to benchmark and evaluate the performance of different rendering engines, develop new rendering algorithms and techniques, optimize rendering parameters, and understand rendering challenges. By enabling more realistic and efficient rendering, advancing research in lighting simulation, and facilitating the development of AI-driven rendering techniques, this dataset has the potential to shape the future of computer graphics and rendering technology.

Specifications Table

**Table:** 

Subject	Computer Graphics and Computer-Aided Design
Specific subject area	3D rendered images for 3D render engine analysis and machine learning.
Data format	Raw
Type of data	RGB Image (.png)
Data collection	The data was collected by generating 63,648 RGB images via Blender 4.0 Cycles/Eevee render engines, featuring Blender's primitive shapes with 13 different materials and 6 lighting setups. Each of them was adjusted with Blender's Color Management preset options for diverse renderings.
Data source location	Hosted on Mendeley Data. Accessible at doi.org/10.17632/c49hdpxvyw.1
Data accessibility	The data is published in Mendeley Data. Repository name: Render Lighting Dataset: A Collection of Rendered Images with Varied Lighting Conditions using Blender Render Engines Data identification number: 10.17632/c49hdpxvyw.1 Direct URL to data: https://data.mendeley.com/datasets/c49hdpxvyw/1

## 1 Value of the Data

• Researchers can use this dataset to develop and test new rendering techniques, including real-time rendering algorithms, by analyzing the interplay of light and material under various conditions.
• The Render Lighting Dataset can be a standard benchmark for machine learning algorithms focused on image recognition, lighting estimation, and material property analysis. Its consistent and controlled variables allow for objective comparison of algorithm performance.
• The inclusion of different materials offers a rich resource for researchers to understand and model the visual properties of materials under diverse lighting conditions.
• The dataset provides a diverse set of images that can be used to train machine learning models to recognize different 3D shapes, lighting conditions, and material textures, which is crucial in computer vision and graphics applications.
• It can be used as an educational tool for students learning about computer graphics, providing hands-on experience with a dataset that demonstrates fundamental principles of lighting and material in rendering.

## 2 Background

Computer graphics have come a long way and are now in high demand for creating computer-generated visuals in movies, advertisements, product designs, and other fields. Render engines play a vital role in turning project ideas into real products. Different engines can produce different results, which raises the question of how to improve them [1]. Blender has two primary render engines, Eevee and Cycles, both of which are great and excel in specific areas. Eevee is faster, while Cycles produces superior quality, highlighting the trade-offs in rendering approaches [2]. Lighting is crucial to determine an object's realism, and improving lighting is a big challenge, especially in 3D rendering engines involving Virtual Reality (VR) and Augmented Reality (AR). To mimic real-world lighting and material accuracy in rendering engines and for object detection and tracking, diverse datasets are critical [3]. This context enriches the significance of the Render Lighting Dataset [4] as a benchmark for evaluating and improving rendering algorithms, catering to the nuanced demands of different rendering engines across various industries.

## 3 Data Description

**Fig. 1:**
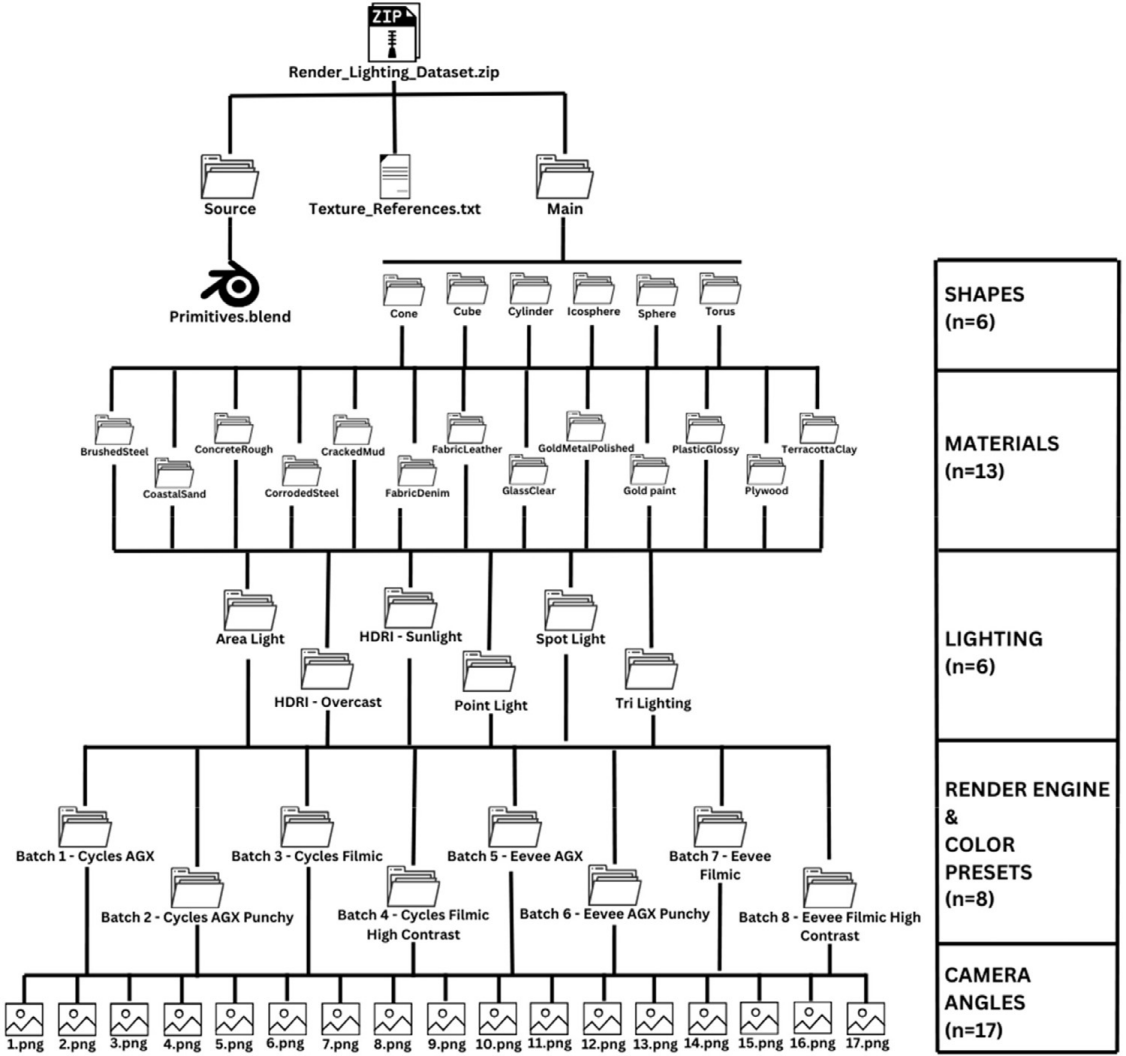
The file structure of the Render Lighting dataset along with the number of folders.

This dataset contains numerous 3D-rendered images of various primitive shapes of Blender under different lighting conditions. Created using Blender's version 4.0 and its Cycles and Eevee Render Engines, we have generated 63,648 RGB images (.png). The dataset includes the source file (.blend file) for easier reproducibility. The rendered images feature Blender's primitive shapes with additional geometry for better texture mapping and UV unwrapping. The dataset offers six different lighting setups to get a better understanding of how different render engines interpret different lighting conditions. Each rendered image has been adjusted using Blender's Color Management panel, with View Transform and Look options tailored to four distinct settings. The dataset also features 17 different camera angles capturing each rendered scene, allowing researchers to explore the interplay between lighting, materials, and geometry from various perspectives. With 13 different materials, researchers can study material properties and visual aesthetics in detail. The dataset has been organized into a detailed file structure, allowing for easy access and use by researchers. Fig. 1 illustrates the file structure of the dataset.

**Fig. 2:**
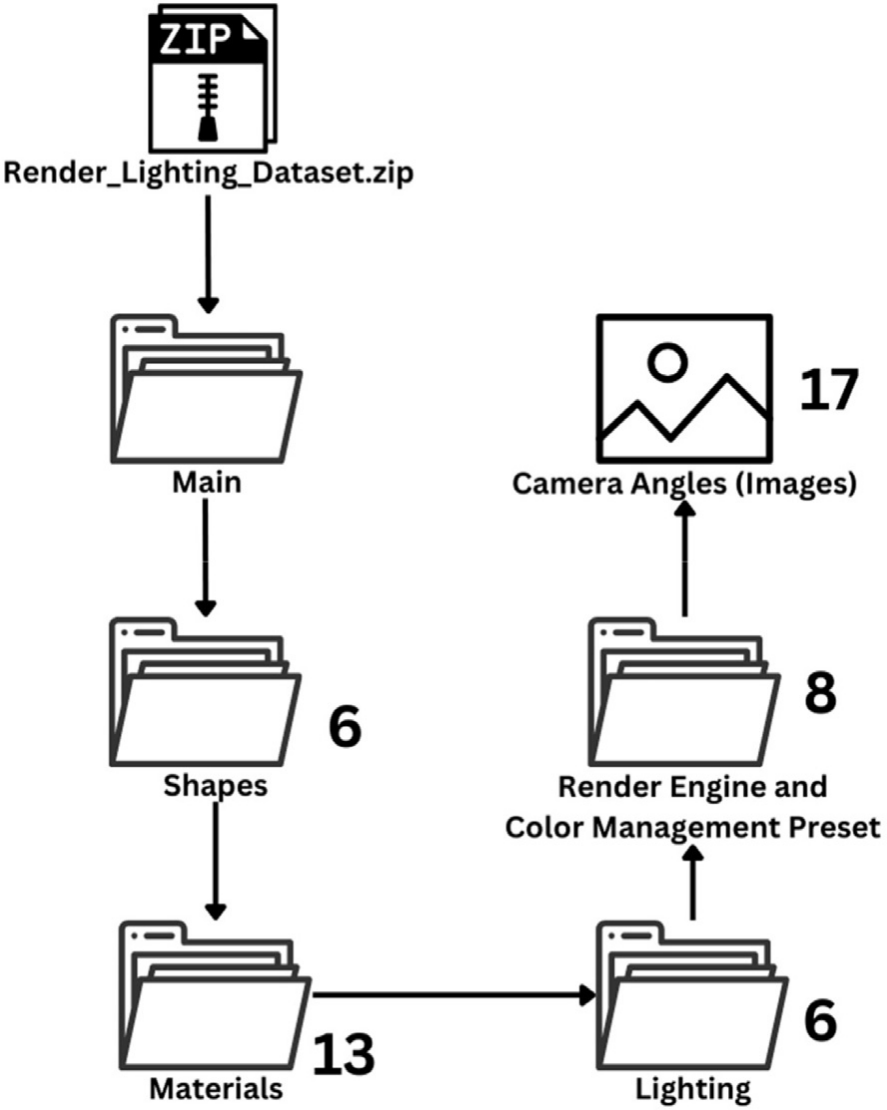
The simplified file structure of the Render Lighting dataset along with the number of folders inside each sub-folder.

The Render Lighting dataset (7.17 GB) is contained within a highly compressed zip file, denominated ‘Render_Lighting_Dataset.zip’, and is composed of two primary folders, namely, Source and Main, and a text file, named ‘Texture_References.txt’. The ‘Texture_References.txt’ file enlists the names of the textures and HDRI utilized in the generation of the dataset, together with the corresponding links from where they were legally obtained. All textures and HDRI used in the dataset are free to use. The ‘Source’ folder encompasses the Blender source file (.blend), which was employed to create the dataset. All the textures and HDRI are embedded within the source file. The main files (images) of the dataset are located in the ‘Main’ folder, which is further organized into several sub-folders, and the images have been renamed, sorted, and categorized inside these folders. There are 6 folders inside the main folder, each representing a shape used in the dataset. These are Cone, Cube (Bevelled), Cylinder (Hollow), Icosphere, Sphere (Subdivided Cube) and Torus. Within each shape folder, there are 13 folders representing the 13 materials that were used for each shape. These materials are BrushedSteel, CoastalSand, CorrodedSteel, CrackedMud, FabricDenim, FabricLeather, GlassClear, GoldMetalPolished, GoldPaint, PlasticGlossy, Plywood, and TerracottaClay. Within each material folder, there are 6 folders representing the lighting conditions used for each shape of each material. These lighting folders are Area Light, HDRI – Overcast, HDRI – Sunlight, Point Light, Spot Light, and Tri Lighting. Within each lighting folder, there are 8 folders representing each batch that was rendered with different render engines and Color management options. These batches are named ‘Batch 1 – Cycles AGX’, ‘Batch 2 – Cycles AGX Punchy’, ‘Batch 3 – Cycles Filmic’, ‘Batch 4 – Cycles Filmic High Contrast’, ‘Batch 5- Eevee AGX’, ‘Batch 6 – Eevee AGX Punchy’, ‘Batch 7 – Eevee Filmic’, and ‘Batch 8 – Eevee Filmic High Contrast’. Within each batch folder, you will find 17 RGB (no alpha) images representing different camera angles for those categories. These images are named from 1 to 17 and have the .png extension. Fig. 2 provides a simplified representation of the file structure.

**Fig. 3:**
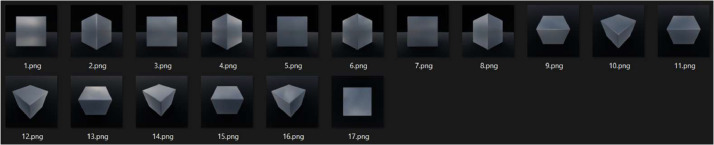
Brushed steel cube rendered with cycles using HDRI overcast lighting and AGX punchy settings.

**Fig. 4:**
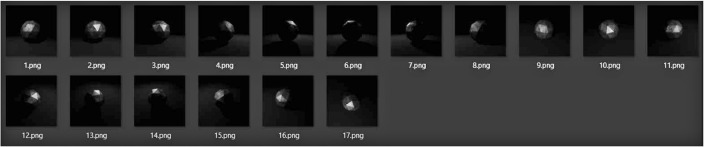
Corroded steel icosphere rendered with Eevee using tri-lighting setup and Filmic settings.

Some samples of the dataset have been shown in Figs. 3 and 4 for each shape in different camera angles, materials, lighting conditions, render engines, and color management options.

## 4 Experimental Design, Materials and Methods

The main objective of this dataset was to create a repository of rendered images using Blender software to assist researchers in enhancing 3D rendering engines. The dataset can also be utilized for various purposes such as machine learning, artificial Intelligence, computer vision, and pattern recognition and detection and can also be utilized in projects dealing with computer-simulated virtual worlds.

### 4.1 Shape design

To create the images, we started by creating shapes and an environment in which to render them. Instead of using default primitive shapes, we edited them to improve texture mapping. The background was a white plane inside a smooth sphere. Table 1 provides a detailed specification of each shape used in the dataset.

**Table 1:** Specifications for each of the primitive shapes used in the dataset.

Shape	Dimension (X, Y, Z)	Bevelled	Subdivided
Cone	2.22 m x 2.22 m x 1.99m	No	No
Cube	2 m x 2 m x 2m	Yes Bevel-Width: 0.05 m Bevel-Segments: 11	No
Cylinder	2 m x 2 m x 2 m Hollowed Inner Dimension: 1.63 × 1.63 × 2m	Yes Bevel-Width: 0.02 m Bevel-Segments: 4	Yes 3 edge rings manually added in the outer and inner center
Icosphere	1.9 m x 2 m x 2m	No	No
Sphere	2.27 m x 2.27 m x 2.27m	No	Yes Cube Subdivided 5 times using subdivision surface modifier
Torus	2.97 m x 2.97 m x 0.989 m Major Radius: 1 m Minor Radius: 0.5	No	Yes 1 time using subdivision surface modifier

**Fig. 5:**
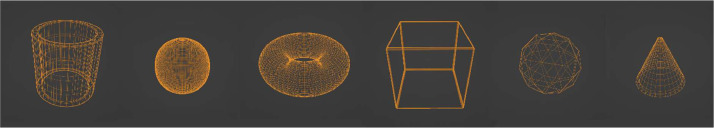
Wireframe view of each shape used in the dataset.

In order to understand the topology of the shapes better, a wireframe view of each shape has been included in Fig. 5.

### 4.2 Lighting setups

**Fig. 6:**
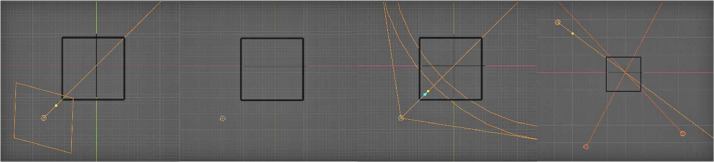
Lighting setup for each light source – Area, Point, Spot, and Tri respectively.

Afterward, we proceeded to arrange the lighting and camera for the shoot. To set up the HDRI, we rotated it positively by 60° in the Z axis and 2 K resolution HDRI maps were used. As for the point light, spotlight, area light, and tri lights, we positioned them in one corner so that one side receives more light than the others. For the light sources that can be adjusted, we aimed them at the center of the object. The point light, spotlight, and area light were all set to a power of 100 W, and a pure white color was used for each of them. The spotlight was given a spot size of 60° and a blend of 0.150 units while the area light was given a size of 2 m. The shadow caustics were disabled, and multiple importance was enabled for all light sources. As for the tri lights, each of them was given a unique intensity and color. The backlight was set to a light blue color (Hex: #D1F7FF) and an intensity of 35 W. The fill light was given a pure white color with an intensity of 25 W, while the Key light was also set to a pure white color with a high intensity of 100 W. The lighting setup for these four non-HDRI light sources can be observed in Fig. 6.

### 4.3 Material design

Next, we prepared the materials for the shapes. We gathered the materials from various online resources that are free to use and set them up utilizing Blender's nodes. Our main focus was on the base color (diffuse) map, roughness map, normal map (OpenGL), displacement map, and metalness map. All the texture maps utilized in the renders are in 2 K resolution. All of the shader setups are similar, however, the shader setup for glass differs slightly due to the way Blender's Cycles and Eevee render engines handle transparent and translucent materials. In Eevee, the blend mode of the glass shader was set to 'Alpha Blend', and the Shadow mode was set to 'Opaque'.

### 4.4 Render engine parameters

In the render engine settings, the screen space refraction and subsurface translucency were turned off, and no bloom or ambient occlusion was used. In eevee, the render sample count was set to 16 with the remaining settings set to default. For Cycles, the render sample count was also set to 16, the noise threshold was set to 0.0100, and denoising was enabled with the denoiser set to 'OpenImageDenoise'. Passes were set to 'Albedo and Normal', and the prefilter was set to 'Accurate' while the rest of the settings were left default. The color management settings were adjusted between View Transform set to AGX and Filmic and Look set to None, Punchy, and High-Contrast respectively. The punchy option is only available with the latest AGX view transform option, so for the filmic view transform, a high-contrast look was used instead as an alternative.

### 4.5 Camera setups

Finally, we adjusted the camera and angles. We used a perspective-type camera with a 24 mm focal length and clip start set to 0.01 and end set to 1000 m. We didn't use any depth of field. To capture the light from different angles, we used 17 different camera angles by adjusting the X-axis and Z-axis rotations while keeping the Y-axis rotation at 0°. A detailed specification of the camera angles used is provided in Table 2.

**Table 2:** Specifications for each camera angle.

Image Name	Rotation (X-Axis) in degrees	Rotation (Y-Axis) in degrees	Rotation (Z-Axis) in degrees
1.png	90	0	0
2.png	90	0	−45
3.png	90	0	−90
4.png	90	0	−135
5.png	90	0	−180
6.png	90	0	−225
7.png	90	0	−270
8.png	90	0	−315
9.png	45	0	0
10.png	45	0	−45
11.png	45	0	−90
12.png	45	0	−135
13.png	45	0	−180
14.png	45	0	−230
15.png	45	0	−270
16.png	45	0	−320
17.png	0	0	0

### 4.6 Data collection

The texture maps and HDRI were collected from PolyHaven, AmbientCG, Texture.com, BlenderKit and Poliigon. The details of which material or HDRI was collected from which platform are given in the Texture_References.txt file within the main zip folder. The Blender 4.0 software was downloaded from the official Blender website (https://www.blender.org/). The images were rendered using these materials and several other adjusted parameters and the raw rendered image was provided in the dataset. A total of 63,648 RGB images (.png) were generated and then renamed and categorized into folders. The source file has been included in the dataset.

## Limitations

The primary limitation of the Render Lighting Dataset is its specific focus on Blender's primitive shapes and the predefined lighting conditions and materials. While offering a broad spectrum for testing and development in 3D rendering, this focus might limit its applicability to scenarios requiring more complex or naturalistic object models and environments. Additionally, the dataset's reliance on Blender 4.0′s rendering engines (Cycles and Eevee) may restrict its utility for researchers working with other 3D rendering software or those interested in cross-platform rendering performance comparisons. These constraints could influence the dataset's generalizability and applicability to a wider range of real-world applications in computer graphics and machine learning.

## Ethics Statement

The authors confirm adherence to the ethical requirements for publication in Data in Brief, ensuring that this work does not involve human subjects, animal experiments, or any data collected from social media platforms.

## Credit Author Statement

**Khandoker Ashik Uz Zaman:** Conceptualization, Methodology, Writing – Original Draft, Formal analysis, Data curation, Rendering, Formatting. **Ashraful Islam:** Supervision, Writing – Reviewing and Editing. **Md Abu Sayed:** Supervision, Reviewing, and Editing.

## Data Availability

Render Lighting Dataset: A Collection of Rendered Images with Varied Lighting Conditions using Blender Render Engines (Original data) (Mendeley Data).
